# CD56bright cells respond to stimulation until very advanced age revealing increased expression of cellular protective proteins SIRT1, HSP70 and SOD2

**DOI:** 10.1186/s12979-018-0136-5

**Published:** 2018-11-28

**Authors:** Lucyna Kaszubowska, Jerzy Foerster, Daria Schetz, Zbigniew Kmieć

**Affiliations:** 1 Department of Histology, Medical University of Gdańsk, Dębinki 1, 80-211 Gdańsk, Poland; 2 Department of Social and Clinical Gerontology, Medical University of Gdańsk, Dębinki 1, 80-211 Gdańsk, Poland; 3 Department of Pharmacology, Medical University of Gdańsk, Dębowa 23, 80-204 Gdańsk, Poland

**Keywords:** NK cells, CD56dim cells, CD56bright cells, Ageing, SIRT1, HSP70, SOD2, Innate immunity, Immunosenescence, Cellular protective proteins

## Abstract

**Background:**

NK cells are cytotoxic lymphocytes of innate immunity composed of: cytotoxic CD56dim and immunoregulatory CD56bright cells. The study aimed to analyze the expression of cellular protective proteins: sirtuin 1 (SIRT1), heat shock protein 70 (HSP70) and manganese superoxide dismutase (SOD2) in CD56dim and CD56bright NK cells of the young, seniors aged under 85 (‘the old’) and seniors aged over 85 (‘the oldest’). We studied both non-stimulated NK cells and cells stimulated by IL-2, LPS or PMA with ionomycin. The expression level of proinflammatory cytokines TNF and IFN-γ was also assessed in NK cell subsets and some relationships between the studied parameters were analyzed.

**Results:**

CD56bright cells showed sensitivity to most of the applied stimulatory agents until very advanced age in regards to the expression of SIRT1 and intracellular HSP70. On the contrary, CD56dim cells, sensitive to stimulation by most of the stimulatory agents in the young and the old, in the oldest lost this sensitivity and presented rather high, constant expression of SIRT1 and HSP70, resistant to further stimulation. With reference to SOD2 expression, CD56dim cells were insensitive to stimulation in the young, but their sensitivity increased with ageing. CD56bright cells were sensitive to most of the applied agents in the young and the old but in the oldest they responded to all of the stimulatory agents used in the study. Similarly, both NK cell subsets were sensitive to stimulation until very advanced age in regards to the expression of TNF and IFN-γ.

**Conclusions:**

CD56bright cells maintained sensitivity to stimulation until very advanced age presenting also an increased expression of SIRT1 and HSP70. CD56dim cells showed a constantly increased expression of these cellular protective proteins in the oldest, insensitive for further stimulation. The oldest, however, did not reveal an increased level of SOD2 expression, but it was significantly elevated in both NK cell subsets after stimulation.

The pattern of expression of the studied cellular protective proteins in ageing process revealed the adaptation of NK cells to stress response in the oldest seniors which might accompany the immunosenescence and contribute to the long lifespan of this group of the elderly.

## Background

Natural killer cells (NK cells) are innate lymphoid cells (ILCs) crucial for immune response against viral infection and tumor cells [1]. They are characterized by the expression of CD56 surface marker and divided into two functionally different subsets based on their cell surface density of CD56. CD56bright cells, less numerous subset (10% of peripheral blood NK cells) are the primary source of NK cell-derived immunoregulatory cytokines, whereas CD56dim cells that constitute the majority of peripheral blood NK cell population (90%) are highly cytotoxic effector cells that can also produce high levels of IFN-γ upon stimulation [2, 3]. Our group [4] and other investigators showed that the absolute number of NK cells increases with age similarly to the ratio of CD3-CD56^dim^ to CD3-CD56^bright^ cells [5–7]. These changes result from the increase of the absolute number of CD56dim NK cells and decline of the total number of CD56^bright^ cells within peripheral blood in the process of ageing [4, 5, 7, 8]. However, detailed characteristics of the alterations of the CD56bright and CD56dim cells in ageing process have not yet been described.

NK cells are classified as part of the innate immune system due to expression of germ-line encoded receptors that do not undergo receptor gene rearrangement to generate antigen-specific receptors like T and B lymphocytes [9]. NK cells can exhibit some features usually associated with adaptive immunity, i.e. they can adapt to changing cellular environment and develop a type of antigen-specific immunological memory [10, 11]. After stimulation by cytokines, e.g. IL-2, IL-12, IL-15 and IL-18 or a target cell challenge they produce a range of cytokines (TNF, IFN-γ, IL-5, IL-10, IL-13, GM-CSF) and chemokines (IL-8, MIP-1α, MIP-1β and RANTES [12–14].

Interleukin 2 is crucial for NK cells’ growth, proliferation and enhancement of their cytolytic function. It activates JAK/STAT pathway and transcription of various genes involved in the regulation of these processes [15, 16]. IL-2 can activate also PI3K-Akt or MEK/ERK pathway resulting in cell survival and cell cycle progression [17]. MEK/ERK pathway was also shown to be necessary for the activation of NK cells, IFN-γ secretion, CD25 and CD69 expression, and enhanced cytotoxic function [18].

Lipopolysaccharide (LPS) is a component of the outer membrane of Gram-negative bacteria recognized by Toll-like receptors 4 (TLR4) [19–21]. These receptors play a pivotal role in innate immunity and are expressed also on the surface of NK cells [22]. TLR4 receptors were also reported to be expressed intracellularly in NK cells of healthy humans [23]. TLR4 binds lipid A, a part of lipopolysaccharide molecule and this interaction activates NF-κB pathway and expression of genes coding for proinflammatory cytokines, e.g. TNF, IL-1, IL-6, GM-CSF and chemokines, e.g. IL-8, RANTES, MIP-1α, MCP-1 [24, 25]. Kanevskiy et al. suggests also the existence of a distinct, TLR4-independent mechanism of a direct action of LPS on NK cells, that leads to their activation and secretion of IFN-γ [26].

Phorbol 12-myristate 13-acetate (PMA) is a protein kinase C (PKC) activator used for a strong and unspecific stimulation of NK cells in combination with ionomycin (Ca^2+^ ionophore A23187), a calcium ion channel-opening antibiotic. PMA mimicks the action of inositol triphosphate (IP3) and increases intracellular cytoplasmic free Ca^2+^ concentration by causing an influx of calcium ions from the extracellular space into the cell [27]. Ca^2+^-PKC pathway is involved in the phosphorylation of STAT4 which binds to promoters of numerous genes, including proinflammatory cytokines, e.g. IFN-γ and TNF. PMA with ionomycin are usually used for short (1–6 h) cell stimulations to induce the expression of cytokines, e.g. IFN-γ [28–30]. However, the combination of these agents is also applied for both short [30] and longer (up to 48 h) cell stimulations to analyze profiles of gene and protein expression [31, 32].

Ageing of the immune system is associated with continuing increase in the proinflammatory status caused by the declining capacity to cope with stress factors and deterioration of regulatory mechanisms concerning cytokine secretion. This process is characterized by increased serum levels of proinflammatory cytokines IL-6 and TNF, raised level of C reactive protein, decreased concentration of anti-inflammatory cytokine IL-10 [33] and is described as inflamm-aging [34]. Immunosenescence is also accompanied by the increase in oxidative stress caused by disparity between the level of reactive oxygen species (ROS) generation and diminished activity of cellular anti-oxidative mechanisms [35–37].

Adaptive stress response protects cells against unfavorable environmental conditions and different types of injuries. Eukaryotic cells evolved networks of signaling pathways to detect and control diverse forms of stress. Among them, chaperons play a key role. They are highly conserved proteins, responsible for preservation of the correct conformation of cellular macromolecules, i.e. proteins, RNA and DNA [38]. Their expression was found to highly correlate with maximum lifespan of vertebrates, indicating higher levels of HSP expression in longer-lived species of mammals and birds [39]. HSP70 play then a pivotal role in the protein quality control system, which is of vital importance to counteracting the ageing process [36].

SIRT1 is a redox-sensitive protein that reveals protective properties mediating an oxidative stress response [40]. It directly deacetylates several transcription factors involved in the regulation of expression of some antioxidant and cellular protective genes, e.g. the FOXO family of transcription factors which promote the expression of stress response genes including SOD2 [41, 42]. SIRT1 promotes mitochondrial biogenesis and upregulates the expression of genes involved in oxidative stress response, i.e. glutathione peroxidase (GPx1), catalase, superoxide dismutases 1 and 2 (SOD1 and SOD2) [43, 44]. Westerheide et al. showed also that in mammalian cells SIRT1 is involved in the regulation of heat shock response as it directly deacetylates HSF1 [45]. Heat shock factor 1 (HSF1) is activated in response to a variety of stresses - including heat shock, hypoxia, misfolded proteins, free radicals and adenosine triphosphate depletion. These two systems of cellular protective proteins function together to protect cells from various stresses, promote survival and extend the lifespan of invertebrates, e.g. *Caenorhabditis elegans* and *Drosophila melanogaster* [40, 46] or small mammals as was shown in experiments on mouse embryonic fibroblasts derived from SIRT1 knockout mice [41].

Recently, the expression of SIRT1, HSP70 and SOD2 in the elderly, including seniors in very advanced age, has been described also in NK cells [4, 47]. However, there are no data about the expression of cellular protective proteins in two subpopulations of NK cells, i.e. CD56dim and CD56bright cells during ageing. Therefore, the aim of our study was to analyze the expression of SIRT1, HSP70 and SOD2 in CD56dim and CD56bright NK cells of the young, seniors under 85 and the oldest seniors aged over 85. The studied cells were non-stimulated or stimulated by IL-2, LPS or PMA with ionomycin to assess the expression level of the analyzed protective proteins. Moreover, the expression of proinflammatory cytokines, i.e. TNF and IFN-γ was also evaluated in the studied NK cell subpopulations in various age groups. Finally, we analyzed the potential relationships between the studied proteins in the process of ageing.

## Material and methods

### Participants

Eighty six volunteers aged between 19 and 94 years (62 women and 24 men) participated in this study. The exclusion criteria included: CRP > 5 mg/L, cancer, autoimmune disease, diabetes, infection, use of immunosuppressors, glucocorticoids or non-steroid anti-inflammatory drugs (NSAID). Absence of dementia was assessed using the “Mini Mental State Examination” and only seniors with the score above 23 points were qualified to the study [48]. Senior volunteers underwent then a geriatric assessment. The Katz’s index of independence in “Activities of Daily Living” (ADL) was used and only seniors with 5–6 points were enrolled to the study [49]. Senior volunteers were recruited among inhabitants of local retirement homes whereas young volunteers were students of Medical University of Gdańsk, Poland. The participants were subdivided into 3 groups including: 31 young subjects referred to as ‘young’ (20.9 ± 0.3 years, range 19–24 years, 22 women and 9 men); 30 seniors aged under 85 referred to as ‘old’ (mean age 75.6 ± 0.9 years, range 65–84 years, 20 women and 10 men) and 25 seniors at the age over 85 referred to as the ‘oldest’ (mean age 88.4 ± 0.5 years, range 85–94 years; 20 women and 5 men). All volunteers signed informed consent and the study received approval from Ethical Committee of Medical University of Gdańsk, Poland (No 225/2010). An immunological characteristics of the study population was described earlier [4].

### Preparation of peripheral blood mononuclear cell cultures

Peripheral blood mononuclear cells (PBMCs) were isolated from venous blood samples collected in tubes with EDTA by conventional ficoll-uropoline density gradient centrifugation. PBMCs were then washed and resuspended in RPMI1640 medium supplemented with 5% FBS, penicillin (100 U/ml) – streptomycin (100 μg/ml) and 2-mercaptoethanol (5 × 10^− 5^ M) (all purchased from SigmaAldrich, Saint Louis, MO, USA). Cells (5 × 10^5^ / 0.5 ml) were cultured for 48 h in the absence (control) or presence of IL-2 (100 U/ml) (BD Biosciences, San Jose, CA, USA), LPS (1 μg/ml) or PMA (50 ng/ml) and ionomycin (500 ng/ml, all purchased from Sigma-Aldrich). PBMCs treated in this way were analyzed for the expression of SIRT1, SOD2 and HSP70 (surface and intracellular). The intracellular expression of TNF and IFN-γ, was studied in PBMCs (5 × 10^5^ / 0.5 ml) cultured in the absence (control) or presence of IL-2 (100 U/ml) (BD Biosciences, San Jose, CA, USA), LPS (1 μg/ml) Sigma-Aldrich, Saint Louis, MO, USA) or PMA (50 ng/ml) and ionomycin (500 ng/ml) (SigmaAldrich, Saint Louis, MO, USA) for 5 h. Simultaneously, Golgi Stop reagent (0.5 μl / well in 0.5 ml of medium, BD Biosciences, San Jose, CA, USA) was added to PBMC cultures (5 × 10^5^ / 0.5 ml) to stop extracellular export of cytokines. Then PBMCs were collected and washed with 1 ml of BD Staining Buffer.

### Staining of surface and intracellular antigens for flow cytometry

PBMCs (2.5 × 10^5^ cells) were aliquoted into flow cytometry tubes and CD3-FITC-conjugated (0.125 μg/ml; clone UCHT1) (BD Biosciences, San Jose, CA, USA) or CD3-PE-Cy7-conjugated (0.125 μg/ml; clone SK7) (BD Biosciences, San Jose, CA, USA), CD56-APC-conjugated (0.6 μg/ml; clone NCAM16.2) (BD Biosciences, San Jose, CA, USA) and Hsp70-PE-conjugated (1 μg/ml; clone N27F34) (Abcam, Cambridge, England) monoclonal antibodies were added for cell surface antigen staining. After 30 min of incubation in the dark at room temperature cells were washed twice with 1 ml of BD Staining Buffer (PBS without Ca^2+^ and Mg^2+^, 1% FBS, 0.09% sodium azide) and resuspended in 0.25 ml of Fixation/Permeabilization Solution for 20 min at 4 °C following manufacturer’s protocol (BD Cytofix/Cytoperm Fixation/Permeabilization Kit). Cells were washed twice with 1 ml of BD Perm/Wash buffer and relevant volumes of MnSOD-FITC-conjugated (1 μg/ml; clone MnS-1) (eBioscience, San Diego, CA, USA), Hsp70-PE-conjugated (1 μg/ml; clone N27F34) (Abcam, Cambridge, England), SIRT1-Alexa Fluor 488 – conjugated (1 μg/ml; clone 19A7AB4) (Abcam, Cambridge, England), TNF-PE-Cy7- conjugated (0.125 μg/ml; clone MAb11) (BD Biosciences, San Jose, CA, USA) or IFN-γ-PE-conjugated (0.125 μg/ml; clone 4S.B3) (BD Biosciences, San Jose, CA, USA) monoclonal antibodies were added for staining of intracellular antigens following the manufacturer’s instructions. After 30 min of incubation in the dark at room temperature cells were washed twice with 1 ml of BD Perm/Wash buffer and resuspended in Staining Buffer prior to flow cytometric analysis. Samples were run on a BD FACSCalibur flow cytometer equipped with argon-ion laser (488 nm) and data were evaluated with BD CellQuest Pro software (BD Biosciences, San Jose, CA, USA) after collecting 10,000 gated events (lymphocytes). Peripheral blood lymphocytes were gated using forward (FSC) and side scatter (SSC) parameters. NK cells were identified in the CD3-negative region based on the expression of CD56 surface marker and defined as CD3-CD56+ cells. NK cell subsets were then discriminated, i.e. CD56dim and CD56bright cells and further analyzed for the frequency of cells expressing the particular cellular protective protein (SIRT1, HSP70, SOD2) or cytokine (TNF and IFN-γ). Relevant isotype controls for both surface and intracellular staining were also used. Results were then presented in two ways, i.e. as percentages of CD56dim and CD56bright cells with the expression of the studied protein (% of positive cells) and mean fluorescence intensity (MFI).

### Statistics

All data are expressed as means ± SEM. Normality of data distribution was analyzed by Shapiro-Wilk test. ANOVA test for normal distribution and Kruskal-Wallis test for non-parametric distribution were used to compare experimental data. The multiple comparisons were performed with Tukey’s post-hoc test for normal distribution and Dunn’s post-hoc test for non–parametric distribution. Paired Student’s t-test for normal distribution and Wilcoxon signed-rank test for non-parametric distribution were used to compare two related samples. Student’s t test for normal distribution and Mann-Whitney U test for non-parametric distribution were used to compare two independent samples. The Spearman correlation coefficient (R) was applied to present the strength of the relationship between variables (Statistica, version 12; Statsoft, Tulsa, OK, USA). Differences or correlations with *p* < 0.05 were considered as statistically significant.

## Results

### Expression of SIRT1 in CD56dim and CD56bright cells

**Fig. 1:**
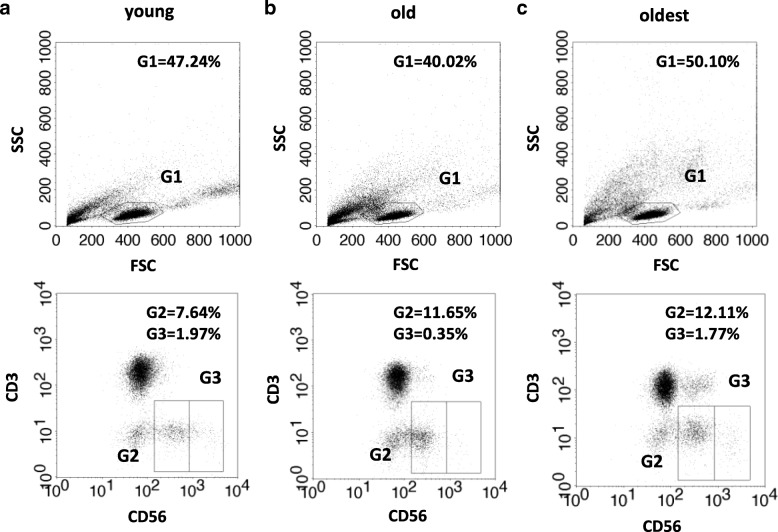
Gating strategy performed for NK cell subsets of the young (**a**), the old aged under 85 (**b**) and the oldest aged over 85 (**c**). Upper dot plots refer to lymphocyte gating - lymphocytes (G1) were gated within PBMC population based on FSC and SSC parameters. Lower dot plots refer to NK cell subsets gating: CD56dim (G2) and CD56bright (G3) cells were discriminated by low vs. high expression of CD56 within NK cell population defined as CD3 negative and CD56 positive cells (CD3-CD56+)

**Fig. 2:**
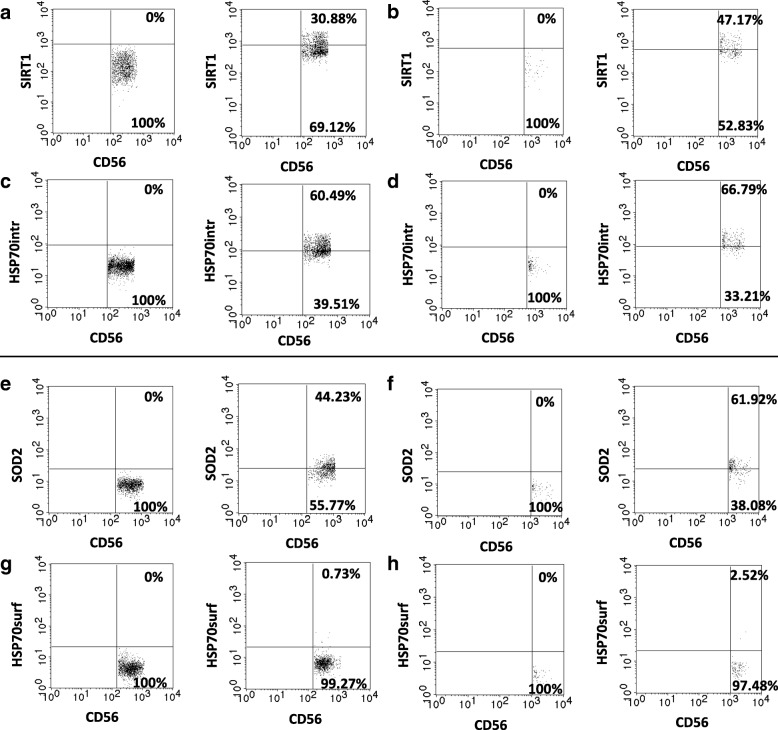
Flow cytometry analysis performed on NK cell subsets. Upper panel: CD3/CD56/SIRT1/HSP70^intracellular^ staining (**a-d**). Lower panel: CD3/CD56/SOD2/HSP70^surface^ staining (**e-h**). **a** SIRT1 staining in CD56dim cells: left - isotype control; right - sample, CD56dim cells expressing SIRT1 were identified in the upper right quadrant. **b** SIRT1 staining in CD56bright cells: left - isotype control; right - sample, CD56bright cells expressing SIRT1 were identified in the upper right quadrant. **c** HSP70^intracellular^ (HSP70intr) staining in CD56dim cells: left - isotype control, right - sample; CD56dim cells expressing intracellular HSP70 were identified in the upper right quadrant. **d** HSP70^intracellular^ (HSP70intr) staining in CD56bright cells: left – isotype control; right sample; CD56bright cells expressing intracellular HSP70 were identified in the upper right quadrant. **e** SOD2 staining in CD56dim cells: left - isotype control, right - sample; CD56dim cells expressing SOD2 were identified in the upper right quadrant. **f** SOD2 staining in CD56bright cells: left - isotype control; right - sample; CD56bright cells expressing SOD2 were identified in the upper right quadrant. **g** HSP70^surface^ (HSP70surf) staining in CD56dim cells: left - isotype control, right - sample; CD56dim cells expressing surface HSP70 were identified in the upper right quadrant. **h** HSP70^surface^ (HSP70surf) staining in CD56bright cells: left - isotype control, right - sample; CD56bright cells expressing surface HSP70 were identified in the upper right quadrant

The gating strategy performed for NK cell subsets of the young, old and the oldest is demonstrated in Fig. 1 and flow cytometry analysis scheme is shown in Fig. 2. Flow cytometry data were analyzed and presented in two ways, i.e. as percentages of CD56dim and CD56bright cells with the expression of the studied protein (% of positive cells) and relative expression, i.e. mean fluorescence intensity (MFI) measured in the analyzed samples.

**Fig. 3:**
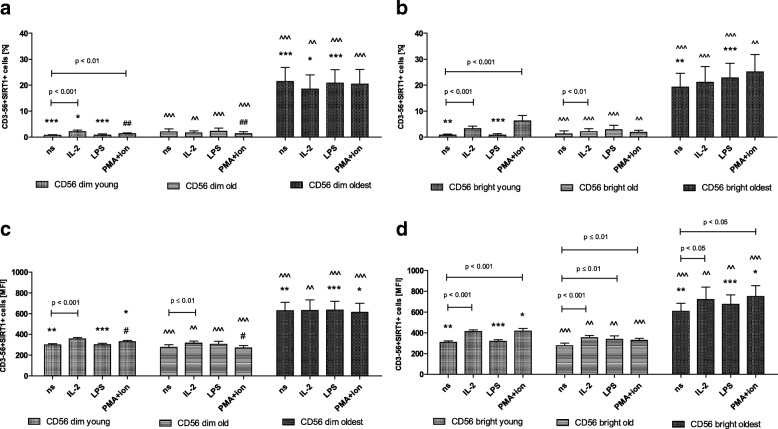
Expression of SIRT1 in non-stimulated and stimulated CD56dim and CD56bright cells of the young, old and the oldest. Data are presented as means ± SEM and show expression of the studied protein in NK cell subsets demonstrated as the percentage of cells with the expression of a particular protein (%) and mean fluorescence intensity (MFI). The same symbols over bars (^#,^ *^,^ ^) denote statistically significant differences between similarly treated CD56dim or CD56bright cells, i.e. respectively non-stimulated or stimulated by IL-2, LPS or PMA with ionomycin compared between: young vs. old (marked with ^#^), young vs. oldest (marked with *) and old vs. oldest (marked with ^). 3 symbols denote *p* < 0.001; 2 symbols denote *p* < 0.01; 1 symbol denotes *p* < 0.05. Horizontal lines above paired bars denote statistically significant differences between respectively CD56dim or CD56bright cells non-stimulated vs. stimulated by IL-2, LPS or PMA with ionomycin within the same age group. **a** Expression of SIRT1 in CD56dim cells (%). **b** Expression of SIRT1 in CD56bright cells (%). **c** Expression of SIRT1 in CD56dim cells (MFI). **d** Expression of SIRT1 in CD56bright cells (MFI)

Flow cytometry data analysis performed in the young and the old (seniors at the age under 85) revealed that the expression of SIRT1 was rather low in non-stimulated CD56dim and CD56bright cells. Both subsets, however, were sensitive to stimulation with IL-2 and PMA with ionomycin, which was particularly observed in the young in the analysis of the percentages of positive cells as well as in relative expression (Fig. 3a-d). CD56bright cells of the old analyzed with the MFI method additionally revealed sensitivity to stimulation by LPS (Fig. 3d). This tendency was found also in the relevant analysis of the percentages of positive cells (Fig. 3b). CD56dim cells of the old were practically insensitive to stimulation except for IL-2 stimulation in MFI analysis, not found in the analysis of percentages (Fig. 3a, c).

Both subsets of NK cells in the young presented a slightly increased level of SIRT1 expression in stimulated cells compared to non-stimulated ones (Fig. 3a-d). However, after comparison of similarly treated CD56dim vs. CD56bright cells, we found it significantly higher in CD56bright cells both without stimulation and upon stimulation with IL-2, LPS or PMA and ionomycin (Table 1). In the old CD56bright cells were more sensitive to stimulation than CD56dim ones (Fig. 3d). However, the expression of SIRT1 in CD56bright cells of the old was higher compared to similarly treated CD56dim cells only upon stimulation with LPS (Table 1).

**Table 1:** Comparison of the analyzed parameters between similarly treated CD56dim and CD56bright cells of the same age group

Parameter	Stimulation type	CD56dim vs. CD56bright					
		young		old		oldest	
		%	MFI	%	MFI	%	MFI
SIRT1	ns	** ↑ bright	–	*** ↑ dim	–	–	–
	IL2	–	** ↑ bright	–	–	–	–
	LPS	*** ↑ bright	–	* ↑ bright	–	–	–
	PMA/ion	–	*** ↑ bright	–	–	–	–
HSP70^intr^	ns	–	–	–	–	–	–
	IL2	* ↑ bright	** ↑ bright	–	–	–	–
	LPS	–	–	–	–	–	–
	PMA/ion	* ↑ bright	** ↑ bright	–	** ↑ bright	–	–
HSP70^surf^	ns	*** ↑ dim	*** ↑ dim	*** ↑ bright	*** ↑ dim	*** ↑ dim	*** ↑ dim
	IL2	–	–	*** ↑ dim	–	* ↑ dim	–
	LPS	*** ↑ bright	* ↑ dim	*** ↑ dim	–	*** ↑ dim	–
	PMA/ion	*** ↑ dim	*** ↑ dim	–	–	–	–
SOD2	ns	–	–	** ↑ dim	–	–	–
	IL2	–	–	–	* ↑ bright	*** ↑ bright	** ↑ bright
	LPS	–	–	–	–	** ↑ bright	–
	PMA/ion	–	–	–	–	–	–
TNF	ns	*** ↑ bright	*** ↑ dim	*** ↑ dim	*** ↑ dim	*** ↑bright	** ↑dim
	IL2	–	*** ↑ dim	–	*** ↑ dim	–	–
	LPS	*** ↑ dim	*** ↑ dim	*** ↑dim	*** ↑ dim	*** ↑ dim	** ↑ dim
	PMA/ion	*** ↑ dim	*** ↑ dim	*** ↑dim	*** ↑ dim	*** ↑ dim	** ↑ dim
IFN-γ	ns	–	–	*** ↑ dim	–	–	–
	IL2	–	–	–	–	–	–
	LPS	–	–	** ↑ dim	–	–	–
	PMA/ion	–	* ↑ dim	–	–	* ↑ bright	–

↑denotes significantly higher expression in dim or bright cells

*'HSP70*^*intr'*^ intracellular HSP70, *'HSP70*^*surf*^' surface HSP70, '*PMA/ion*' PMA + ionomycin

*** *p* < 0.001; ** *p* < 0.01; * *p* < 0.05

Non-stimulated CD56dim and CD56bright cells of the oldest seniors showed significantly increased expression of SIRT1 compared with the young and the old (Fig. 3a-d). There were not observed any significant differences between non-stimulated and stimulated CD56dim and bright cells in the analysis of the percentages of positive cells (Fig. 3a, b). However, the analysis of the relative expression revealed in the oldest increased level of SIRT1 upon stimulation with IL-2 and PMA with ionomycin in CD56bright cells (Fig. 3d), not observed in CD56dim cells (Fig. 3c).

### Expression of HSP70^intracellular^ in CD56dim and CD56bright cells

**Fig. 4:**
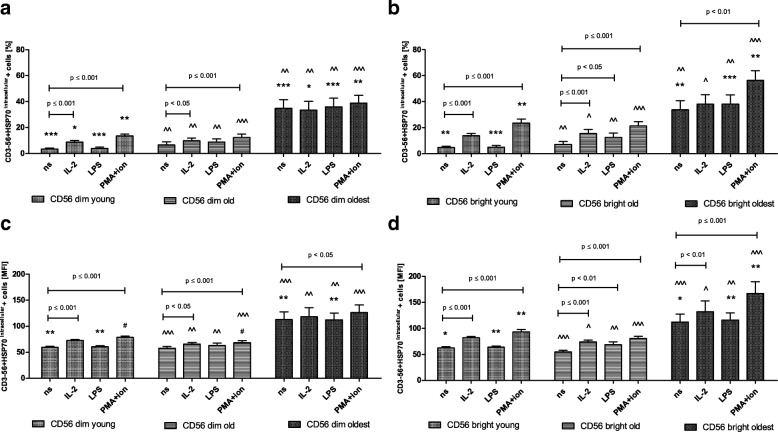
Expression of HSP70^intracellular^ in non-stimulated and stimulated CD56dim and CD56bright cells of the young, old and the oldest Data are presented as means ± SEM and show expression of the studied protein in NK cell subsets demonstrated as the percentage of cells with the expression of a particular protein (%) and mean fluorescence intensity (MFI). The same symbols over bars (^#,^ *^,^ ^) denote statistically significant differences between similarly treated CD56dim or CD56bright cells, i.e. respectively non-stimulated or stimulated by IL-2, LPS or PMA with ionomycin compared between: young vs. old (marked with ^#^), young vs. oldest (marked with *) and old vs. oldest (marked with ^). 3 symbols denote *p* < 0.001; 2 symbols denote *p* < 0.01; 1 symbol denotes *p* < 0.05. Horizontal lines above paired bars denote statistically significant differences between respectively CD56dim or CD56bright cells non-stimulated vs. stimulated by IL-2, LPS or PMA with ionomycin within the same age group. **a** Expression of HSP70^intracellular^ in CD56dim cells (%). **b** Expression of HSP70^intracellular^ in CD56bright cells (%). **c** Expression of HSP70^intracellular^ in CD56dim cells (MFI). **d** Expression of HSP70^intracellular^ in CD56bright cells (MFI)

The pattern of HSP70^intracellular^ expression in CD56dim and CD56bright cells resembled the expression of SIRT1 in the young and the old. It was rather low in non-stimulated cells, however, both subsets of NK cells were sensitive to stimulation by IL-2 and PMA with ionomycin (Fig. 4a-d). CD56bright cells of the old presented the highest sensitivity and responded to all three types of stimulation observed in the analysis of the percentages of positive cells as well as relative expression (Fig. 4b, d). Although both subsets of NK cells in the young and the old presented an increased level of intracellular HSP70 expression upon stimulation (Fig. 4a-d), it was significantly higher in CD56bright cells compared to similarly treated CD56dim cells of the young observed in both types of analysis (Table 1). In NK cells of the old, the similar phenomenon was found only after stimulation with PMA and ionomycin when the relative expression was analyzed (Table 1).

Non-stimulated CD56dim and CD56bright NK cells of the oldest seniors showed significantly increased expression of intracellular HSP70 compared with the young and the old (Fig. 4a-d). There were no significant differences between non-stimulated and stimulated CD56dim and CD56bright cells in the analysis of the percentages of positive cells, except for CD56bright cells stimulated with PMA and ionomycin (Fig. 4b). The analysis of the relative expression showed, however, the increased level of HSP70^intracellular^ in both CD56dim and CD56bright cells treated with PMA and ionomycin and also in CD56bright cells upon stimulation with IL-2 (Fig. 4c, d).

### Expression of HSP70^surface^ in CD56dim and CD56bright cells

**Fig. 5:**
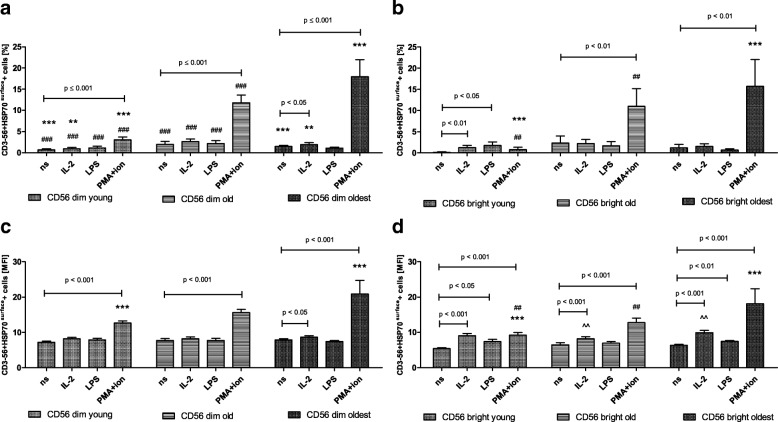
Expression of HSP70^surface^ in non-stimulated and stimulated CD56dim and CD56bright cells of the young, old and the oldest Data are presented as means ± SEM and show expression of the studied protein in NK cell subsets demonstrated as the percentage of cells with the expression of a particular protein (%) and mean fluorescence intensity (MFI). The same symbols over bars (^#,^ *^,^ ^) denote statistically significant differences between similarly treated CD56dim or CD56bright cells, i.e. respectively non-stimulated or stimulated by IL-2, LPS or PMA with ionomycin compared between: young vs. old (marked with ^#^), young vs. oldest (marked with *) and old vs. oldest (marked with ^). 3 symbols denote *p* < 0.001; 2 symbols denote *p* < 0.01; 1 symbol denotes *p* < 0.05. Horizontal lines above paired bars denote statistically significant differences between respectively CD56dim or CD56bright cells non-stimulated vs. stimulated by IL-2, LPS or PMA with ionomycin within the same age group. **a** Expression of HSP70^surface^ in CD56dim cells (%). **b** Expression of HSP70^surface^ in CD56bright cells (%). **c** Expression of HSP70^surface^ in CD56dim cells (MFI). **d** Expression of HSP70^surface^ in CD56bright cells (MFI)

HSP70^surface^ revealed a quite different pattern of expression in NK cells. It was relatively low in non-stimulated cells, however, sensitive to stimulation by PMA with ionomycin in CD56dim cells of all age groups (Fig. 5a, c). The highest sensitivity to stimulation presented CD56dim cells of the oldest seniors as they increased expression of HSP70^surface^ after stimulation by both IL-2 and PMA with ionomycin in both types of analysis (Fig. 5a, c). However, CD56bright cells were even more sensitive compared to CD56dim cells as they responded to all three types of stimulation in the group of the young and the oldest when the relative expression was measured (Fig. 5d). In the same analysis  CD56bright cells of the old responded only to stimulation by IL-2 and PMA with ionomycin (Fig. 5d). NK cells generally showed increased level of surface HSP70 expression upon stimulation. In most cases it was significantly higher in CD56dim cells compared to similarly treated CD56bright cells as we observed in all age groups and in both types of analysis (Table 1).

### Expression of SOD2 in CD56dim and CD56bright cells

**Fig. 6:**
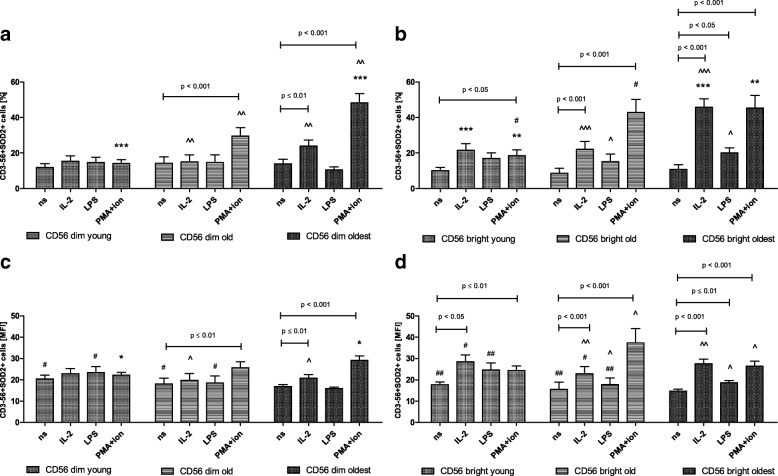
Expression of SOD2 in non-stimulated and stimulated CD56dim and CD56bright cells of the young, old and the oldest Data are presented as means ± SEM and show expression of the studied protein in NK cell subsets demonstrated as the percentage of cells with the expression of a particular protein (%) and mean fluorescence intensity (MFI). The same symbols over bars (^#,^ *^,^ ^) denote statistically significant differences between similarly treated CD56dim or CD56bright cells, i.e. respectively non-stimulated or stimulated by IL-2, LPS or PMA with ionomycin compared between: young vs. old (marked with ^#^), young vs. oldest (marked with *) and old vs. oldest (marked with ^). 3 symbols denote *p* < 0.001; 2 symbols denote *p* < 0.01; 1 symbol denotes *p* < 0.05. Horizontal lines above paired bars denote statistically significant differences between respectively CD56dim or CD56bright cells non-stimulated vs. stimulated by IL-2, LPS or PMA with ionomycin within the same age group. **a** Expression of SOD2 in CD56dim cells (%). **b** Expression of SOD2 in CD56bright cells (%). **c** Expression of SOD2 in CD56dim cells (MFI). **d** Expression of SOD2 in CD56bright cells (MFI)

There were no significant differences in the expression of SOD2 between non-stimulated and stimulated CD56dim cells of the young. Then, the sensitivity to stimulation of these cells increased with ageing and in the group of seniors below 85 years CD56dim cells became sensitive to stimulation by PMA with ionomycin observed in both types of analysis (Fig. 6a, c). CD56bright cells of the young were sensitive to stimulation by PMA and ionomycin in the analysis of the percentages of positive cells (Fig. 6b) and additionally to IL-2 observed when the analysis of relative expression was performed (Fig. 6d). Similarly, the old revealed in both types of analysis comparable to the young sensitivity of CD56bright cells to the stimulation and responded to IL-2 and PMA with ionomycin (Fig. 6b, d).

NK cell subsets of the oldest seniors presented the highest sensitivity to stimulation, i.e. by IL-2 and PMA with ionomycin in CD56dim cells (Fig. 6a, c) and all three types of stimulation in CD56bright cells found in both types of analysis (Fig. 6b, d). Although the subsets of NK cells, except for CD56dim cells of the young, increased level of SOD2 expression upon most types of stimulation (Fig. 6a-d), it was significantly higher in CD56bright cells of the oldest stimulated with IL-2 and LPS compared to similarly treated CD56dim cells (Table 1). In the group of the old this phenomenon was observed only in CD56bright cells stimulated by IL-2 in MFI analysis (Table 1).

### Expression of TNF in CD56dim and CD56bright cells

**Fig. 7:**
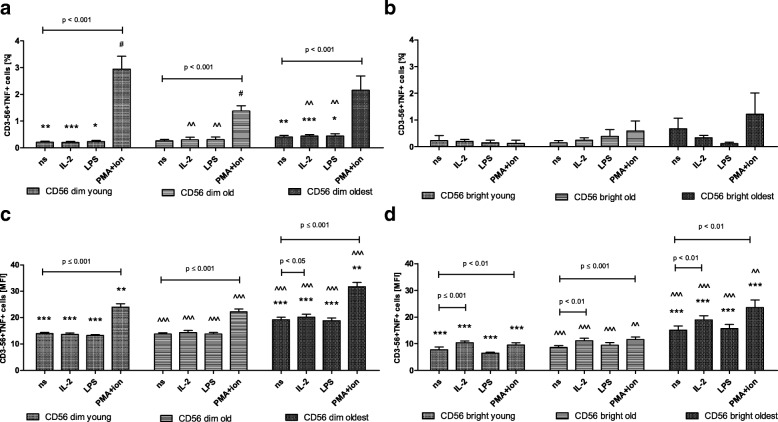
Expression of TNF in non-stimulated and stimulated CD56dim and CD56bright cells of the young, old and the oldest Data are presented as means ± SEM and show expression of the studied protein in NK cell subsets demonstrated as the percentage of cells with the expression of a particular protein (%) and mean fluorescence intensity (MFI). The same symbols over bars (^**#,**^ *^,^ ^) denote statistically significant differences between similarly treated CD56dim or CD56bright cells, i.e. respectively non-stimulated or stimulated by IL-2, LPS or PMA with ionomycin compared between: young vs. old (marked with ^#^), young vs. oldest (marked with *) and old vs. oldest (marked with ^). 3 symbols denote *p* < 0.001; 2 symbols denote *p* < 0.01; 1 symbol denotes *p* < 0.05. Horizontal lines above paired bars denote statistically significant differences between respectively CD56dim or CD56bright cells non-stimulated vs. stimulated by IL-2, LPS or PMA with ionomycin within the same age group. **a** Expression of TNF in CD56dim cells (%). **b** Expression of TNF in CD56bright cells (%). **c** Expression of TNF in CD56dim cells (MFI). **d** Expression of TNF in CD56bright cells (MFI)

The expression of TNF in non-stimulated cells was significantly higher in both CD56dim and CD56bright cells of the oldest compared to the young and the old (Fig. 7a-d). CD56dim cells of all age groups appeared to be sensitive to stimulation by PMA with ionomycin observed both in the analysis of the percentages of positive cells and the relative expression (Fig. 7a, c). Additionally, CD56dim cells of the oldest seniors showed sensitivity to stimulation by IL-2 found in MFI analysis (Fig. 7c). CD56bright cells of all age groups were also sensitive to these two types of stimulation when the analysis of the relative expression was performed (Fig. 7d). Although CD56bright cells appeared to be more sensitive to stimulation, CD56dim cells revealed higher expression of TNF compared to CD56bright ones when similarly treated cells were compared within the analyzed age group (Table 1).

### Expression of IFN-γ in CD56dim and CD56bright cells

**Fig. 8:**
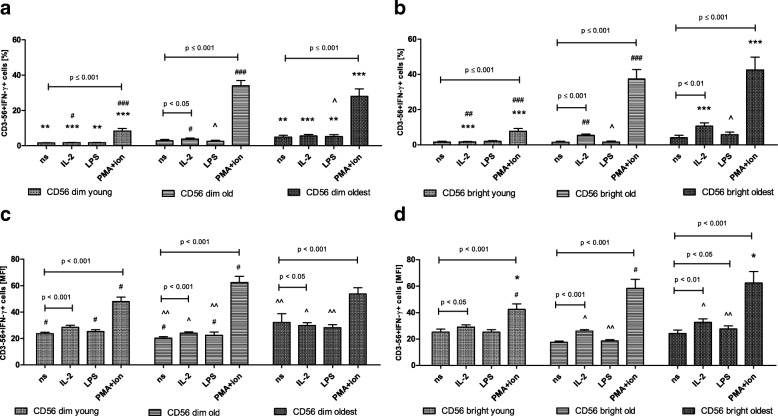
Expression of IFN-γ in non-stimulated and stimulated CD56dim and CD56bright cells of the young, old and the oldest Data are presented as means ± SEM and show expression of the studied protein in NK cell subsets demonstrated as the percentage of cells with the expression of a particular protein (%) and mean fluorescence intensity (MFI). The same symbols over bars (^#,^ *^,^ ^) denote statistically significant differences between similarly treated CD56dim or CD56bright cells, i.e. respectively non-stimulated or stimulated by IL-2, LPS or PMA with ionomycin compared between: young vs. old (marked with ^#^), young vs. oldest (marked with *) and old vs. oldest (marked with ^). 3 symbols denote *p* < 0.001; 2 symbols denote *p* < 0.01; 1 symbol denotes *p* < 0.05. Horizontal lines above paired bars denote statistically significant differences between respectively CD56dim or CD56bright cells non-stimulated vs. stimulated by IL-2, LPS or PMA with ionomycin within the same age group. **a** Expression of IFN-γ in CD56dim cells (%). **b** Expression of IFN-γ in CD56bright cells (%). **c** Expression of IFN-γ in CD56dim cells (MFI). **d** Expression of IFN-γ in CD56bright cells (MFI)

The pattern of IFN-γ expression in CD56dim cells of all age groups resembled the expression of TNF in these cells. However, both CD56dim and CD56bright cells of all studied age groups were sensitive to stimulation by IL-2 and PMA with ionomycin what was clearly documented by the relative expression and also to some extent by the analysis of the percentages of positive cells (Fig. 8a-d). The most sensitive to stimulation appeared to be CD56bright cells of the oldest seniors which responded to all types of the applied stimulations shown in the analysis of the relative expression (Fig. 8d). The analysis of the percentages of positive cells revealed the predominance of CD56bright vs. CD56dim cells in expression of IFN -γ upon stimulation with PMA and ionomycin observed in the oldest (Table 1). The similar analysis performed in the old and the young showed however higher expression of IFN-γ rather in CD56dim cells compared to CD56bright cells (Table 1).

### Relationships between the analyzed parameters in CD56dim and CD56bright NK cells

The flow cytometric analysis provided multitude of data what allowed to reveal some relationships between the analyzed parameters. Interestingly, they were observed both in CD56dim and CD56bright cells and corresponded to some extent to those reported earlier by our group in the total NK cell population [47]. Very high positive correlations between SIRT1 and HSP70^intracellular^ expression in all studied set-ups of CD56dim cells, i.e. non-stimulated or treated with IL-2, LPS or PMA with ionomycin, were noted (Table 2). Similarly, high and very high positive correlations between these parameters were observed within CD56bright cells (Table 3). Low positive correlations were found between the expression of SIRT1 and: (i) TNF in all applied conditions, (ii) IFN-γ in all conditions except for stimulation with PMA + ionomycin in both CD56dim and CD56bright cells and additionally without LPS stimulation in CD56bright cells, (iii) SOD2 in samples stimulated with IL-2 in CD56bright cells and PMA with ionomycin in CD56dim cells and (iv) HSP70^surface^ in cells stimulated with IL-2 within the population of CD56 bright cells (Tables 2 and 3).

**Table 2:** Correlation analysis of the study population performed for CD56dim cells

CD56dim						
Parameter	Stimulation type	SOD2	SIRT1	HSP70^intr^	HSP70^surf^	TNF
SIRT1	none	ns				
	IL-2	ns				
	LPS	ns				
	PMA/ion	0.221				
HSP70^intr^	none	ns	0.968			
	IL-2	ns	0.948			
	LPS	ns	0.973			
	PMA/ion	0.296	0.929			
HSP70^surf^	none	0.646	ns	ns		
	IL-2	0.740	ns	0.230		
	LPS	0.716	ns	ns		
	PMA/ion	0.652	ns	ns		
TNF	none	ns	0.391	0.423	0.244	
	IL-2	ns	0.266	0.337	0.406	
	LPS	ns	0.372	0.385	0.301	
	PMA/ion	0.413	0.413	0.393	0.426	
IFN-γ	none	ns	0.407	0.453	ns	0.295
	IL-2	ns	0.425	0.481	ns	0.245
	LPS	0.265	0.370	0.431	0.371	0.333
	PMA/ion	ns	ns	ns	ns	ns

All values are presented as statistically significant (*p* < 0.05) Spearman’s correlation coefficients (R). ‘ns’ denotes statistically not significant

*'HSP70*^*intr*^' intracellular HSP70, '*HSP70*^*surf*^' surface HSP70, *'PMA/ion'* PMA + ionomycin

**Table 3:** Correlation analysis of the study population performed for CD56bright cells

CD56bright						
Parameter	Stimulation type	SOD2	SIRT1	HSP70^intr^	HSP70^surf^	TNF
SIRT1	none	ns				
	IL-2	0.235				
	LPS	ns				
	PMA/ion	ns				
HSP70^intr^	none	ns	0.888			
	IL-2	0.240	0.958			
	LPS	ns	0.966			
	PMA/ion	0.263	0.820			
HSP70^surf^	none	0.576	ns	ns		
	IL-2	0.880	0.296	0.322		
	LPS	0.750	ns	ns		
	PMA/ion	0.739	ns	ns		
TNF	none	ns	0.409	0.391	0.263	
	IL-2	0.234	0.271	0.298	0.415	
	LPS	ns	0.424	0.430	0.220	
	PMA/ion	0.263	0.236	0.411	0.302	
IFN-γ	none	ns	0.310	0.274	ns	ns
	IL-2	ns	0.261	0.306	0.248	0.221
	LPS	0.254	ns	0.319	0.296	0.414
	PMA/ion	ns	ns	ns	ns	0.302

All values are presented as statistically significant (*p* < 0.05) Spearman’s correlation coefficients (R). ‘ns’ denotes statistically not significant

*'HSP70*^*intr*'^ intracellular HSP70, '*HSP70*^*surf'*^ surface HSP70, *'PMA/ion*' PMA + ionomycin

The expression of HSP70^intracellular^ revealed relationships similar to these of SIRT1. Low positive correlations were found between the expression of this protein and: (i) TNF in all applied conditions, (ii) IFN-γ in all conditions except for stimulation with PMA + ionomycin, (iii) SOD2 in samples stimulated with IL-2 in CD56bright cells and PMA + ionomycin in CD56dim and CD56bright cells, and (iv) HSP70^surface^ in cells stimulated with IL-2 in both CD56dim and CD56bright cells (Tables 2 and 3).

The moderate to high correlations between the expression of HSP70^surface^ and SOD2 in all variants of the applied conditions were observed in both CD56dim and CD56bright cells in the whole study group. HSP70^surface^ expression revealed also some lower correlations with other parameters, i.e.: (i) TNF in all applied conditions in both CD56dim and CD56bright cells and (ii) IFN-γ in CD56 bright cells stimulated with IL-2 and in both CD56dim and CD56bright cells stimulated with LPS (Tables 2 and 3). The expression of TNF correlated with: (i) SOD2 in cells stimulated with IL-2 in CD56bright cells and in both CD56dim and CD56bright cells stimulated by PMA with ionomycin (ii) IFN-γ in non-stimulated cells and cells treated with IL-2 and LPS in CD56dim cells and in all variants of stimulation in CD56bright cells. Intracellular IFN-γ correlated positively with the expression of SOD2 in both CD56dim and CD56bright cells treated with LPS (Tables 2 and 3).

### Relationships between the age and the analyzed parameters studied in CD56dim and CD56bright NK cells

Some of the analyzed parameters correlated with age. Remarkably, the expression of SIRT1 showed positive correlations with age in all studied variants, except for the cells stimulated with IL-2 in both CD56dim and CD56bright cells. HSP70^intracellular^ revealed similar to SIRT1 positive correlations in CD56bright cells. Intracellular HSP70 in CD56dim cells correlated positively with age only in non-stimulated cells and upon stimulation with LPS (Table 4). The expression of TNF revealed low to moderate positive correlations with age in all applied experimental conditions in both CD56dim and CD56bright cells. In CD56bright cells HSP70^surface^ correlated positively with age in non-stimulated cells and cells stimulated with LPS and PMA with ionomycin. The expression of HSP70^surface^ in CD56 dim cells showed the positive correlation with age only in cells treated with PMA and ionomycin. IFN-γ correlated positively with age in cells upon stimulation with IL-2 and PMA with ionomycin in CD56bright cells, but not in CD56dim cells (Table 4).

**Table 4:** Correlation analysis of the study population performed for CD56dim and CD56bright cells (age vs. the analyzed parameters)

Compared parameter							
Parameter	Stimulation type	SOD2	SIRT1	HSP70^intr^	HSP70^surf^	TNF	IFN-γ
CD56dim							
Age	none	−0.229	0.289	0.295	ns	0.451	ns
	IL-2	ns	ns	ns	ns	0.499	ns
	LPS	ns	0.409	0.399	ns	0.534	ns
	PMA/ion	0.219	0.263	ns	0.470	0.324	ns
CD56bright							
Age	none	−0.228	0.281	0.226	0.239	0.554	ns
	IL-2	ns	ns	ns	ns	0.518	0.241
	LPS	ns	0.417	0.391	0.277	0.621	ns
	PMA/ion	ns	0.235	0.345	0.421	0.439	0.355

All values are presented as statistically significant (*p* < 0.05) Spearman’s correlation coefficients (R). ‘ns’ denotes statistically not significant

*'HSP70*^*intr*^' intracellular HSP70, *'HSP70*^*surf'*^ surface HSP70, '*PMA/ion*' PMA + ionomycin

## Discussion

Data obtained in the presented study for CD56dim and CD56bright cells correspond to our observations performed earlier on the total NK cell population [47]. Thus, similarly to the previous study, the non-stimulated CD56dim and CD56bright cells of the oldest seniors presented significantly higher expression of SIRT1 compared to seniors under 85 and the young. Correspondingly, results obtained for HSP70^intracellular^ expression were very similar to that of SIRT1. CD56dim cells appeared to be insensitive (SIRT1) or nearly insensitive (HSP70^intracellular^) to the stimulation in the group of the oldest seniors on the contrary to CD56bright cells which in the same age group responded to stimulation by IL-2 and PMA with ionomycin. In the group of seniors under 85 CD56bright cells were also more sensitive to stimulation than CD56dim cells in regard to the expression of both studied cellular protective proteins. In the young both NK cell subsets appeared to be equally sensitive to stimulation. These results may suggest that generally more sensitive to activation CD56bright cells might be involved in rather inducible cellular stress response and this activity seems to be sustained until very advanced age, associated with the increased level of protective proteins in the oldest seniors. The more numerous CD56dim cells [50] were resistant to further stimulation but presented, similarly to CD56bright cells, higher basic expression of cellular protective proteins, i.e. SIRT1 and HSP70. Thus, in the total NK cell population CD56dim cells seem to overshadow the activity of more sensitive to stimulation but much less numerous CD56bright cells [4, 47]. Although both subsets of NK cells presented an increased expression level of SIRT1 and HSP70^intracellular^ after stimulation in the population of the young and the old, nevertheless, after comparison of similarly treated CD56dim vs. CD56bright cells, we found this expression significantly higher in CD56bright cells, with no significant differences observed in the oldest.

Very high correlations between SIRT1 and HSP70^intracellular^ proteins observed in both CD56dim and CD56bright cells might suggest the presence of regulatory loops involved in the control of their expression. SIRT1 was earlier found to stimulate the expression of HSP70 by deacetylation of HSF1 (heat shock factor 1) [45]. This signaling pathway might explain the strong correlation between the analyzed proteins involved in the cellular adaptive response [51]. We observed also the similar correlations in our earlier studies [4, 47].

Although some changes in the level of the expression of cellular protective proteins in ageing were reported, i.e. HSP70 in granulocytes [52], monocytes and lymphocytes [53, 54] or SIRT1-SIRT7 in PBMCs [55], no other study has systematically investigated the effect of ageing on the expression of SIRT1, both forms of HSP70 and SOD2 in two major subpopulations of NK cells.

It is well known that superoxide dismutases are cellular protective enzymes scavenging superoxide generated by mitochondria. The mitochondrial SOD2 is the main antioxidant enzyme that protects cells from oxidative stress [56]. The age-related expression of this dismutase in various human cell types was investigated in skeletal muscle cells [57] and PBMCs [58]. SOD2 expression was analyzed also by our group in ageing human NK cells [4, 47].

The studies performed on skeletal muscle cells of subjects at different age showed the significant increase in SOD2 activity in persons aged between 76 and 85 years as compared to the young (17–25 year-old group) [57] whereas no differences in the levels of SOD2 mRNA were found between young (25 ± 2 y) and older men (61 ± 2 y) [58]. Similarly to the results of these studies the experiments performed on freshly isolated NK cells demonstrated significantly increased SOD2 expression in both CD56dim and CD56bright cells of the oldest seniors compared to the young and the elderly below the age of 85 [4]. In the present study CD56bright cells showed higher sensitivity to stimulation compared to CD56dim cells in the young and both groups of the elderly. Interestingly, NK cell subsets of the oldest appeared to be the most sensitive to stimulation, with a predominance of CD56bright cells, which responded all three types of stimulation. Thus, we have identified a novel feature of NK cell ageing process: the sensitivity to stimulation resulting in the expression of SOD2 increases with age in both NK cell subsets, although with some prevalence of CD56bright cells. Correspondingly to the expression of both SIRT1 and intracellular HSP70, after comparison of similarly treated CD56bright vs. CD56dim cells within the same age group, higher SOD2 expression was found mostly in CD56bright cells.

We also report age-related differences in the expression of HSP70^surface^ in NK cell subsets. Extracellular HSPs act as signaling molecules that activate the immune system in response to stress [59]. The expression of HSP70^surface^ in CD56bright cells was more sensitive to stimulation compared to CD56dim cells similarly to HSP70^intracellular^. However, on the contrary to HSP70^intracellular^, when the expression of HSP70^surface^ was compared within the same age group in both studied NK cell subsets stimulated in the same way, it appeared that CD56dim cells presented higher expression compared to CD56bright cells.

It has been previously noted that extracellular HSPs do not contain a signaling sequence for their secretion via the classic ER-Golgi pathway and they likely use an alternative mechanism of the translocation across the plasma membrane [60]. We could not verify the origin of this protein to find whether it is a membrane form of HSP70 produced by NK cells or extracellular HSP70 bound to TLR receptors on the surface of NK cells. The commercially available antibodies are able to recognize only HSPs bound to TLRs on the cell surface rather than the integrated with the cell membrane form of HSP70 [61].

Interestingly, we observed a high correlation between SOD2 and surface HSP70 expression both in CD56dim and CD56bright cells which was found also in freshly isolated cells [4] and in NK cell cultures [47]. The relationship between surface HSP70 and SOD2 is difficult to explain since we were not able to distinguish between membrane and surface form of HSP70. Other authors in studies performed on PAECs (pulmonary artery endothelial cells) reported interactions between cytosolic stress-inducible HSP70 and SOD2 to maintain this enzyme in an import-competent, unfolded conformation crucial for its transport into the mitochondria [62, 63]. Further studies on transfected PAECs identified HSP70-binding site in SOD2 molecule essential for the interaction of both molecules [64]. However, these findings described the interactions between SOD2 and intracellular form of HSP70. The relationships between these two particular proteins in the previously studied by our group total population of NK cells were rather low and found only in cells stimulated by IL-2 or PMA with ionomycin [47]. Those results, however, were similar to the present study in regards to CD56bright cells as in CD56dim cells the low positive correlation between HSP70^intracellular^ and SOD2 was found only upon stimulation by PMA with ionomycin.

We also observed that the level of HSP70^surface^ expression was many times lower than that of HSP70^intracellular^. These data may indicate that the studied cells were not involved in enhanced inflammatory response. It has been previously suggested that extracellular HSP70 can induce proinflammatory cytokine production [65, 66]. Further studies revealed, however, rather anti-inflammatory properties of extracellular HSP70 and its downregulating effect on the production of proinflammatory cytokines [67–69]. In our studies the expression levels of proinflammatory cytokines (TNF and IFN-γ) were rather low, comparable with these present in non-stimulated cells analyzed shortly after blood sample collection [4]. The highly increased expression of HSP70^surface^ in both CD56dim and CD56bright cells was observed in both groups of seniors only after stimulation with PMA and ionomycin, a stronger stimulatory agent compared to other used in the study.

Then we found that the expression of TNF in non-stimulated and stimulated CD56dim and CD56bright cells was significantly higher in the oldest compared to the young and the old. The process of ageing is characterized by an elevated proinflammatory status, accompanied by increased levels of different inflammatory mediators in apparently healthy people, e.g. CRP, extracellular HSP70, IL-1, IL-6, TNF [70]. Interestingly, both subsets of NK cells of the oldest seniors appeared to be the most sensitive populations to stimulation as was shown in the analysis of the relative expression of these cytokines. The same method of analysis revealed in the old and the young higher sensitivity to stimulation in CD56bright cells compared to CD56dim cells. However, when a comparison of TNF expression between similarly treated CD56dim vs. CD56bright cells within the same age group was performed, a higher expression was observed in CD56dim cells because of its higher basal level in these cells. It is an interesting observation as, generally, CD56bright cells are thought to be the major source of cytokines and CD56dim cells are rather specialized for cytotoxic function [71]. However, upon target cell recognition CD56dim cells could become also more efficient producers of cytokines (e.g. TNF and IFN-γ) than CD56bright NK cells [13]. The process of cytokine secretion by CD56dim cells may be additionally potentiated by exogenous cytokines [72].

IFN-γ presented a slightly different pattern of expression. In CD56dim cells it resembled the expression of TNF. Although both NK cell subsets were sensitive to stimulation by IL-2 and PMA with ionomycin in all age groups, the highest sensitivity to stimulation was observed in CD56bright cells of the oldest. Analogously, the comparison of similarly treated CD56dim vs. CD56bright cells performed within the same age group revealed higher expression of IFN-γ in CD56bright cells. The similar analysis performed in the young and the old showed, however, a predominance of CD56dim cells. These results corresponded to our previously obtained data for freshly isolated cells [4]. CD56bright cells act as immunomodulatory cells in NK cell population but their number decreases with age [73]. Data obtained in our studies might indicate a presence of a compensatory mechanism that maintains the immunoregulatory function of NK cells until very advanced age.

Our results correspond also to some extent with data obtained by De Maria and coworkers [14]. They showed that CD56dim cells could produce IFN-γ two hours after activation and then the amount of cells with the expression of intracellular IFN-γ increased within 4–8 h and afterwards decreased to be non-detectable 16 h after stimulation. On the contrary, CD56bright cells started IFN-γ production later than 16 h after stimulation [14]. These data describing IFN-γ expression in NK cells at short intervals after stimulation resemble our data obtained for the young and the old whereas data on the expression of IFN-γ at later intervals after stimulation seem to correspond to our data obtained for the stimulated CD56bright cells of the oldest. These observations correspond also with our results obtained for freshly isolated NK cells; i.e. short stimulation corresponds with higher expression of IFN-γ in CD56dim cells of the young and longer stimulation corresponds with relatively higher expression of IFN-γ in CD56bright cells of both groups of seniors compared to CD56dim cells [4]. Based on these results we assume that both ageing and NK cell stimulation might influence the level of IFN-γ expression in CD56dim and CD56bright cells. Unfortunately, De Mario et al. did not mention the age of the volunteers involved in the study which might be crucial to compare theirs and ours results. The Italian group, however, underlined a different role of two subsets of NK cells, i.e. involvement of more numerous CD56dim cells in rapid (within hours) immunological reactions typical of frontline innate responses and an immunoregulatory role of CD56bright cells which started to produce cytokines at later intervals. CD56bright NK cells are less numerous but their role may be crucial for the maintenance of the immune homeostasis in healthy ageing [14, 73].

The expression of TNF and IFN-γ correlated positively with SIRT1 and HSP70^intracellular^ in both CD56dim and CD56bright cells. The expression of TNF is under control of NF-κB, a transcription factor involved in the regulation of the inflammatory response [74, 75]. The relationships between SIRT1 activity and TNF secretion were reported in experiments carried out on human monocyte-macrophage cell line MonoMac6. These results showed that activators of SIRT1 downregulated and its inhibitors upregulated release of proinflammatory mediators, such as TNF and IL-8, via NF-κB signaling pathway [76] which might explain the relationships found in our study. The similar relationships were also observed in transgenic SIRT1 mice. They were protected from hepatic inflammation caused by high-fat diet with modest overexpression of SIRT1 which reduced expression of proinflammatory cytokines such as TNF and IL-6 [77].

IFN-γ expression is under control of different than TNF transcription factors, i.e. NFAT, PKC-theta, cJUN / AP-1 [78–80]. However, NF-κB site was also identified within the promoter region of IFN-γ gene demonstrating that NF-κB may be involved in the molecular mechanisms controlling gene transcription of IFN-γ in human T lymphocytes [78] which could directly explain the relationships between SIRT1 and IFN-γ observed in our studies. This signaling pathway was then confirmed in other studies performed on human NK-92 cells [81, 82].

Surface HSP70, despite a rather low expression, revealed some correlations with TNF in all applied conditions both in CD56dim and CD56bright cells observed also in stimulated NK cells cultured in vitro [47]. As we did not analyze the origin of surface HSP70 (intracellular vs. extracellular), we could only hypothesize the reasons for these correlations. Supposing the extracellular origin, HSPs can act as indicators of the stress conditions and prime the other cells, principally of the immune system, to suppress the propagation of the injury [59]. In this case, the binding of extracellular HSP70 to TLRs on the surface of NK cells can stimulate the secretion of proinflammatory cytokines synthesized by these cells, i.e. TNF and IFN-γ. The similar positive correlations between extracellular HSP70 and TNF were observed by Krause and colleagues in plasma samples of the elderly (mean age 63.4 ± 4.4 years) in their studies on insulin resistance [83]. In other studies extracellular HSP70 was found to stimulate IFN-γ production in NK cells, however, dendritic cell-NK cell crosstalk with direct cell-cell contact was crucial to initiate this process [84].

Most of the analyzed parameters correlated with age both in CD56dim and CD56bright cells, i.e. the expression of cellular protective proteins (SIRT1 and HSP70^intracellular^), TNF and partly HSP70^surface^ on the contrary to IFN-γ which correlated with age in CD56bright cells only. These results correspond to our previous data concerning NK cells [47] and results of the other groups, e.g. the increase in the expression of SIRT1 or HSP70^intracellular^ was observed in serum samples [85] or PBMCs [54] of the elderly, respectively. Similarly, an increase in the concentrations of TNF and IFN-γ in sera of the elderly was found in our earlier studies [86] and also was reported by other groups [87].

## Conclusions

The results of our study provide some novel observations concerning the expression of cellular protective proteins: SIRT1, HSP70 and SOD2 in human CD56dim and CD56bright NK cells in regards to the process of ageing. SIRT1 and HSP70 expression appeared to be constantly increased in CD56dim cells of the oldest seniors, which, on the contrary to CD56bright cells, were practically insensitive to further stimulation. CD56bright cells also presented an increased expression of these cellular protective proteins in the oldest but, additionally, they also maintained sensitivity to stimulation until very advanced age. In the young and the old both NK cell subsets showed lower than in the oldest expression level of the studied protective proteins but responded to stimulation by most of the applied agents with some prevalence both in the sensitivity and expression level of CD56bright cells. The oldest seniors did not present an increased level of SOD2 expression but it was significantly elevated in both NK cell subsets after stimulation. The sensitivity to stimulation increased with age and was the highest in the oldest with some predominance of CD56bright cells in this process.

The stimulation of NK cells resulted also in higher expression of surface HSP70 and proinflammatory cytokines, i.e. TNF and IFN-γ. Both CD56dim and CD56bright cells were sensitive to most of the applied types of stimulation until the advanced age. The level of TNF expression after stimulation was higher in CD56dim cells rather than in CD56bright cells and increased with age. Similarly CD56dim cells of the young and the old revealed higher expression of IFN-γ after stimulation. It changed, however, in the oldest who revealed higher expression of IFN-γ in stimulated by PMA and ionomycin CD56bright cells.

Our results clearly show that both subpopulations of NK cells, i.e. CD56dim and CD56bright cells, play different roles in the process of ageing. CD56bright cells seem to be involved in inducible stress response as they can respond to stimulation until very advanced age and simultaneously increase, similarly to CD56dim cells, the basal level of cellular protective proteins SIRT1 and HSP70^intracellular^ in the oldest seniors. CD56dim cells represent both inducible stress response after stimulation in the young and seniors under 85 and rather constant adaptive response in the oldest with higher basal levels of cellular protective proteins insensitive to further stimulation. In conclusion, our study revealed the adaptation of NK cells to stress response in the oldest seniors which might accompany the process of immunosenescence and contribute to the long lifespan of this group of the elderly.

## Abbreviations

ADL: Activities of daily living
CRP: C reactive protein
ERK: Extracellular signal-regulated kinase
FOXO: Forkhead transcription factor class O
GM-CSF: Granulocyte macrophage colony - stimulating factor
HSF: Heat shock factor
HSP: Heat shock protein
MCP: Macrophage chemoattractant protein
MFI: Mean fluorescence intensity
MIP: Macrophage inflammatory protein
MKK: Mitogen - activated protein kinase kinase
MMSE: Mini mental state examination
NF-κB: Nuclear factor kappa-light-chain-enhancer of activated B cells
NSAID: Non-steroid anti-inflammatory drug
PGC: peroxisome proliferator - activated receptor gamma coactivator
PKC: Protein kinase C
PMA: Phorbol myristate acetate
RANTES: Regulated on activation, normal T cell expressed and secreted
SIRT: Sirtuin
SOD: Superoxide dismutase
STAT: Signal transducer and activator of transcription
TLR: Toll-like receptor
